# Bilingualism contributes to resilience in distinct ADRD syndromes

**DOI:** 10.1002/alz.093201

**Published:** 2025-01-03

**Authors:** Jonathan Tablante, Lawren VandeVrede, Emily W. Paolillo, Eugenie Mamuyac, Stephanie M Grasso, Nina Dronkers, Kaitlin B. Casaletto, Jessica Deleon

**Affiliations:** ^1^ Memory and Aging Center, UCSF Weill Institute for Neurosciences, University of California, San Francisco, San Francisco, CA USA; ^2^ Memory and Aging Center, UCSF Weill Institute for Neurosciences, University of California San Francisco, San Francisco, CA USA; ^3^ University of Texas, Austin, TX USA; ^4^ University of California, Berkeley, Berkeley, CA USA; ^5^ Memory & Aging Center, Department of Neurology, University of California in San Francisco, San Francisco, CA USA

## Abstract

**Background:**

Previous studies on the effects of bilingualism on Alzheimer’s disease and related disorders (ADRD) are mixed, and there remains an unclear role of bilingualism in preventing the onset of ADRD (resistance) and in mitigating its effects in the presence of changes related to aging or neuropathology (resilience). Furthermore, the effects of bilingualism may differ depending on the underlying neuropathology, and this remains unexplored.

**Methods:**

In this study, we leveraged a cohort of 484 patients (404 monolingual and 80 bilingual speakers) with Frontotemporal Lobar Degeneration‐Modified Clinical Dementia Rating scale (FTLD CDR) box scores, a measure of clinical disease severity, and plasma neurofilament light chain (NfL) levels, a marker of neurodegeneration, at one or more timepoints. The patients were classified into four groups (healthy controls, Alzheimer’s disease (AD), 4‐repeat (4R) tau, and TAR DNA‐binding protein 43 (TDP‐43)). A total of 302 participants (62% of the cohort) were classified into the four groups based on autopsy‐confirmed neuropathology, while the remaining 182 participants (38% of the cohort) were classified based on predicted pathology. We used linear mixed‐effects models to estimate the longitudinal change in FTLD CDR box scores.

**Results:**

We found that bilingual speakers with AD pathology had a slower rate of change in FTLD CDR box scores compared to monolingual speakers (P = 0.003) when controlled for NfL levels as a proxy of neurodegeneration. This effect was not seen in healthy controls or in patients with tau or TDP pathology.

**Conclusions:**

These results point towards a protective, disease‐specific effect of bilingualism in patients with AD‐related neurodegenerative disease, supporting a role of bilingualism in resilience to ADRD. A strength of this study is its exploration of the effects of bilingualism in a largely autopsy‐confirmed cohort of patients with distinct ADRD neuropathologies. The study may explain the mixed findings in previous studies of bilingualism’s effects, emphasizing the need to explore the effect of bilingualism within specific ADRD subtypes. This may clarify the mechanism by which bilingualism modulates the course of ADRD and contributes to cognitive and brain resilience.

